# Causative Factors, Clinical Manifestations, and Therapeutic Strategies for Irritable Bowel Syndrome

**DOI:** 10.7759/cureus.58728

**Published:** 2024-04-22

**Authors:** Khushi Anand, Mahalaqua Nazli Khatib

**Affiliations:** 1 Medicine, Jawaharlal Nehru Medical College, Datta Meghe Institute of Higher Education and Research, Wardha, IND; 2 School of Epidemiology and Public Health, Jawaharlal Nehru Medical College, Datta Meghe Institute of Higher Education and Research, Wardha, IND

**Keywords:** psychological, been, illness, treatment, clinical, bowel, gastrointestinal, patients, symptoms, ibs

## Abstract

Abdominal distress and irregular bowel movements are the hallmarks of irritable bowel syndrome (IBS), a chronic functional gastrointestinal illness (FGID). It is typified by recurring abdominal discomfort brought on by bowel movements or changes in pattern. Mind-body treatments have gained popularity recently as a way to manage IBS because of the role of the brain-gut axis. In addition to offering a helpful guide for identifying alternate diagnoses in patients exhibiting symptoms similar to IBS, this review attempts to offer an evidence-based solution to these perplexing problems. The etiology, diagnostic standards, and treatments for IBS will be summed up in this review, along with a summary of the available data supporting innovative digital medicines for these two illnesses. This brief study will give an overview of the pathophysiology, clinical characteristics, and treatment strategies of post-infectious irritable bowel syndrome (PI-IBS). In this study, we offer thorough methods for therapeutic therapy and talk about the possible contribution of psychological stress to pathophysiology. Additionally, to help with the introduction and suitability of these patient therapies, we offer a comprehensive review and meta-analysis of randomised controlled trials (RCTs) investigating the effectiveness of exclusion diets (low FODMAP and gluten-free diets, etc.) in IBS.

## Introduction and background

Irritable bowel syndrome (IBS) presents a significant burden on public health, affecting between 5% and 10% of ordinarily healthy individuals at any given time. Its prevalence exhibits notable demographic patterns, disproportionately affecting young adult females. However, beyond its physiological manifestations, IBS is intricately linked with psychosocial comorbidities. Anxiety, depression, and stress are frequently observed alongside IBS, exacerbating symptoms and complicating its management. The association between IBS and psychosocial factors underscores the need to understand the condition's pathogenesis comprehensively. Individuals with these comorbidities often experience more severe symptoms, highlighting the interconnectedness of mental and physical health in IBS patients. This emphasises the importance of further research into the underlying mechanisms of IBS, including its relationship with psychosocial factors, to develop more effective treatment strategies and interventions for managing the condition.

Nonetheless, it is well known that abnormal brain-gut connections result in visceral hypersensitivity, abnormal CNS dispensation, and abnormalities in motility. Other less repeatable processes could be immune system and mucosal dysfunction, gut microbiota changes, and genetic correlations. Unless there are alarming symptoms like weight loss, rectal bleeding, or a family history of celiac disease or inflammatory bowel disease, most patients can be analyzed based on their clinical history and the restricted and prudent use of investigations. After a diagnosis, treating the patient with empathy is essential, as it can lessen symptoms, enhance quality of life, and save medical costs. Soluble fiber, antispasmodic medications, dietary modifications, and patient education on the illness are the cornerstones of treatment. Additional medicines, such as intestinal secretagogues, minimally absorbed antibiotics (chosen based on predominant bowel habits), central neuromodulators, medications acting on opioid or 5-HT receptors, and psychiatric therapies, are often saved for patients with more severe symptoms. Over the past ten years, a robust pipeline of innovative medications has been under development due to an increased understanding of the pathophysiology of IBS [1].

IBS is a persistent and recurrent functional gastrointestinal condition that affects 9-23% of people globally. IBS patients frequently receive referrals to gastroenterology, endure a range of tests, use a variety of medications, miss work, and generally lead miserable lives. The etiology of IBS remains incompletely comprehended and appears complex. Numerous pathogenetic variables can be significant, even if they are not always present in every case and might combine in different ways. An example of a typical clinical manifestation of IBS is discomfort or soreness in the abdomen that is alleviated by feces, along with a change in stool shape. Numerous things, including food and mental stress, might worsen the symptoms. An early diagnosis of IBS is necessary for therapy to provide sufficient symptom relief (diarrhea, constipation, pain, and discomfort). Diagnosing IBS using a particular test or structural anomaly is impossible. Unless the symptoms are deemed abnormal, they are made via principles created on medical symptoms, such as Rome criteria. The present gold customary for IBS diagnosis is the Rome Criteria IV. The Rome IV Criteria is a diagnostic guideline for classifying functional gastrointestinal disorders, including IBS. It provides a standardized framework for healthcare professionals to identify and diagnose these conditions based on specific symptom criteria, such as abdominal pain and changes in bowel habits. These criteria help ensure consistency in diagnosis and facilitate research and treatment of conditions like IBS. IBS sufferers need a comprehensive approach to treatment. While some individuals benefit significantly from non-pharmacological therapy, others must be treated with medication. The pathogenesis, diagnostic standards, and treatments for IBS will be summed up in this review [2].

Due to similar pathophysiologic mechanisms, such as autonomic dysfunction and sensitization of peripheral and brain pain pathways, fibromyalgia and IBS are sometimes co-diagnosed. Estimates suggest that up to 70% of individuals with IBS may also have at least one other functional gastrointestinal disorder, such as functional dyspepsia or functional bloating. In addition to having more severe symptoms and mental health comorbidities, patients with co-diagnoses also have a lower quality of life. Despite significantly influencing central pain syndromes and autonomic dysregulation, the importance of mind-body treatments has not been adequately explained to patients with co-diagnoses. This innovative narrative review evaluates the evidence that is currently available and the application of mind-body therapy in treating IBS and fibromyalgia [3].

The brain-gut axis disorder IBS has several pathophysiological mechanisms, such as abnormal colonic motility, bile acid metabolism, neurohormonal regulation, immunological dysfunction, changed gut secretory features, and alterations in the epithelial barrier. This article examines the mechanisms, safety, and effectiveness of drugs used to treat IBS based on the findings of phase III studies. Particularly for the treatment of pain, efficient treatment is still desperately needed. However, secretion, motility, and a non-absorbable antibiotic are currently the main targets of therapies for IBS bowel dysfunction [4]. IBS symptoms include altered bowel habits and persistent stomach discomfort that lasts longer than six months. The degree of stomach pain and the necessity of seeking medical attention are related. Several gastrointestinal and non-gastrointestinal variables can contribute to the perception of sickness in IBS, but a bidirectionally disturbed gut-brain relationship is critical to the condition's pathophysiology. Mapping these variables into a multidimensional clinical profile helps treat IBS-related abdominal discomfort [5].

Today's pharmaceutical therapies may not be well accepted and sometimes only partially relieve symptoms. Moreover, gastrointestinal symptom relief does not necessarily improve IBS patients' quality of life. According to existing cure strategies, brain-gut behavior therapy (BGBT) should be utilized in addition to currently available IBS treatments and randomized controlled trials. It has been revealed that BGBT improves symptoms, functioning, quality of life, and patient satisfaction. Patient time constraints, costs, and a lack of gastrointestinal psychologists with the required expertise restrict access to BGBT.

Furthermore, referrals could be limited even in cases where they are available since mental health professionals know that BGBT differs from psychotherapy solutions for common mental health conditions. This study comprehensively examines various aspects of IBS, including its underlying biology, burden on individuals, existing treatment gaps, and the potential for emerging digital therapies to address these unmet needs. By synthesizing existing evidence, the study provides insights into the efficacy and suitability of digital interventions for managing IBS symptoms. Recommendations derived from this analysis aim to guide healthcare providers in effectively integrating these interventions into patient care, ultimately improving outcomes and quality of life for individuals living with IBS [6].

Linaclotide is an oral, once-daily, first-in-class guanylate cyclase-C receptor agonist (GC-CA) for the indicative treatment of individuals with moderate-to-severe irritable bowel syndrome with leading constipation (IBS-C). By creating uniform criteria for patient monitoring of IBS-C patients receiving linaclotide, this review seeks to streamline and improve clinical procedures [7]. As a prevalent, unpleasant, and sometimes incapacitating gastrointestinal ailment, IBS lacks a good medicinal or nutritional intervention. Several psychological therapies have been developed and validated in the last ten years. According to two recently completed multiple-site experiments by two investigation teams in the UK and the USA, cognitive behavioral therapy (CBT) is undoubtedly the most effective. Although CBT effectively treats refractory IBS patients, little is known about why this is the case and who benefits most from it. Additionally, because it is typically only available in tertiary care settings, its value proposition may only be protected if additional healthcare savings accompany increased self-management. Efforts to optimize CBT and lessen the influence of IBS on public well-being necessitate a systematic approach to improving CBT's effectiveness and delivery methods, such as integrating digital treatments into primary care [8].

A common functional gastrointestinal illness (FGID), IBS, has been mistakenly labeled psychogenic. While many IBS patients have psychiatric problems as comorbidities, there is not nearly enough data to classify IBS as exclusively psychogenic. Evidence that supports the pure psychogenic explanation of IBS and implies that this condition is a microbe has surfaced recently. Studies highlight microbial alterations in the gut microbiota of IBS patients, alongside immune system dysregulation and disturbances in gut-brain communication. These findings suggest a complex interplay of physiological factors, challenging the pure psychogenic explanation of IBS. As a result, the Rome IV Committee's desire to remove the word "functional" and refer to these conditions as "gut-brain interaction disorders" as opposed to "brain-gut interaction disorders" highlights the role that the gut plays in pathogenesis rather than the brain. Rome IV introduces the concept of a comprehensive clinical profile, considering factors such as overlap, severity, psychological issues, physiological dysfunction, biomarkers, and the diagnostic category of FGID. The goal of this strategy is to pinpoint clinical variability as well as the complexity of pathophysiology and its treatment. One significant new paradigm change is the identification of biological variables in the pathophysiology of IBS. The pathophysiology of peptic ulcer disease progressed from psychological causes to acid reflux disease to Helicobacter pylori infection, which is analogous to that progression. Treatment techniques focusing on the various IBS subtypes' many pathogenic pathways might soon change how the illness is managed [9].

Growing evidence points to dysregulated protease activity as a potential contributing factor to many disorders, even if their exact origins remain unclear. Enzymes called proteases cut other proteins, and their activity is often highly controlled. However, the balance between proteases and their inhibitors frequently upsets during illness, changing the substrate cleavage's temporal and spatial regulation. Chemical methods have been extensively used in evaluating protease levels in normal physiology and illness. Although certain drawbacks exist, these instruments have significantly improved the field [10]. Growing data indicate that psychological variables, rather than healthcare-seeking behavior (brain-gut dysfunction), are responsible for indications in a subgroup of FGID patients. In some situations, intestinal illness may be a secondary cause of psychiatric issues (gut-brain imbalance). To determine if brain-gut and gut-brain syndromes are comparable to IBS and FD, prospective population-based research in FGIDs other than these conditions is required. The lack of FGID phenotyping based on gut-brain versus brain-gut origins in treatment trials may be crucial to determining the actual effectiveness of the interventions. Future studies must identify biological processes that might explain the connection between FGIDs and psychological variables. However, encouraging information is beginning to surface on the brain-gut-immune-microbe axis [11].

The pathophysiology of functional gastrointestinal disorders is thought to be significantly influenced by gut-host-microbe interactions. The most compelling data suggests practical dyspepsia and IBS can arise in susceptible people after acute gastroenteritis. Recent research utilizing 16S rRNA-based microbiota profiling has consistently identified dysbiosis in individuals with IBS, characterized by quantitative and qualitative alterations in mucosal and fecal samples. These changes encompass reduced microbial diversity alongside shifts in the relative abundance of specific bacterial taxa and functional pathways, such as carbohydrate metabolism and short-chain fatty acid production. Such dysbiosis may contribute to IBS symptoms through disruptions in gut barrier function, immune activation, and alterations in visceral sensitivity. This intricate interplay between dysbiosis and IBS underscores the potential therapeutic implications of targeting the gut microbiota to alleviate symptoms and improve patient outcomes. According to the theory, aberrant microbiota triggers innate immune responses in the mucosa, increasing the permeability of the epithelium, triggering nociceptive sensory pathways, and disrupting the enteric nervous system. The microbiome is already a target for therapy, even while we wait for significant discoveries in this area. Although most have small sample sizes and inadequate designs, the controlled studies on non-absorbable antibiotics, prebiotics, probiotics, synbiotics, and diet modification show promise. In this paper, the authors assess the efficacy of microbiota-directed therapies and critically examine existing theories on the pathogenetic role of microbiota in FGID. The authors also offer treatment recommendations for altering the gut microbiota in individuals with IBS [12].

## Review

Search methodology

Eligibility Criteria

All original research on IBS, as well as review publications, met the qualifying requirements. Reviews covered the fundamental ideas behind IBS and its many forms, symptoms, diagnoses, and effects on mental health. Additional exclusion standards did not exist.

Literature Search Strategy

**Figure 1:**
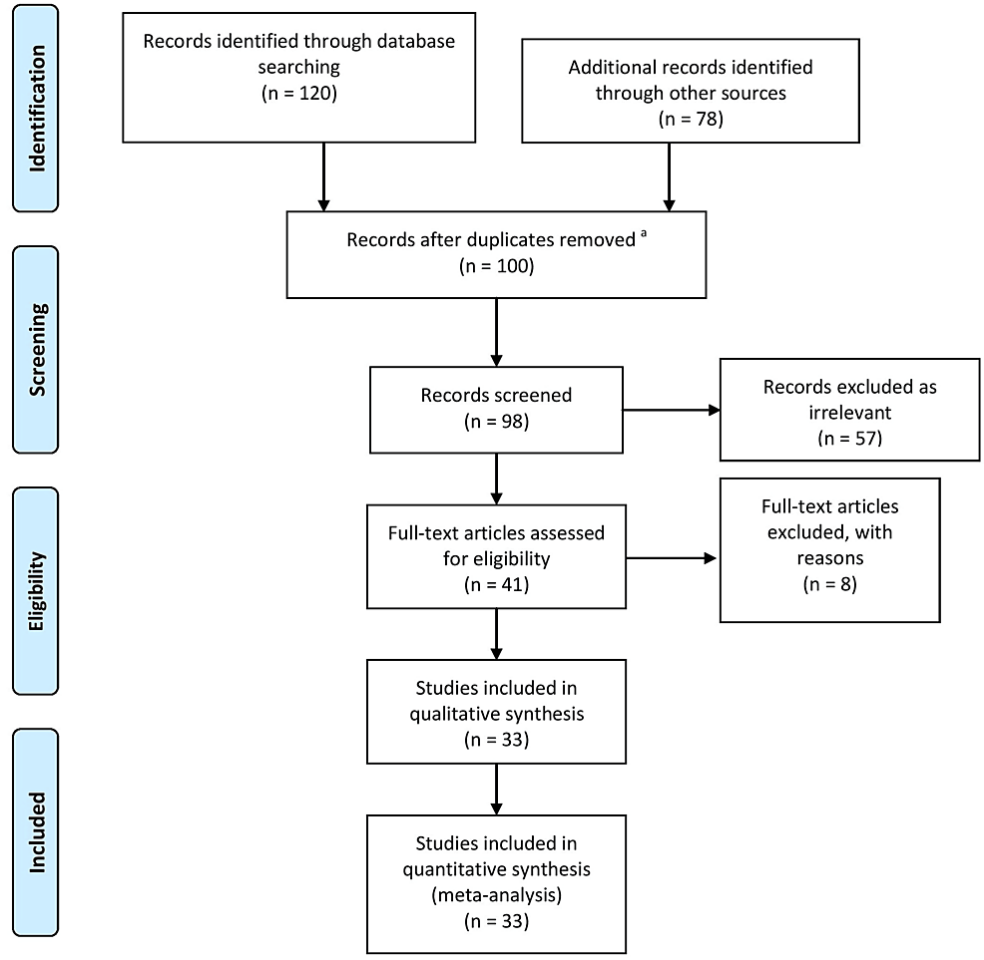
PRISMA flow diagram for screening and selecting articles related to irritable bowel syndrome

Each author did one literature search. We did a literature search using the electronic version of the PubMed database. Published works spanning from 2013 to 2023 were among those that were searched for. Additional records were identified from other sources. Using the keywords "IBS," "FGID," "synbiotics," "IBS patients," "IBS symptoms," "illness," and "treatments," a literature search was carried out. They were coupled with adjuncts of "AND" OR “to examine specific article subtopics (Figure 1).

Clinical presentation

Diagnosis is a very crucial part of clinical presentation. IBS has been separated into three categories: undifferentiated IBS, IBS with variable patterns of diarrhea and constipation, and IBS with diarrhea and discomfort. Even though the precise pathophysiology of IBS is unknown, the condition is complicated and involves both environmental and host factors. Personalized care is necessary for the complex management of people with this illness. Following patient education, nutritional guidance, and stress management, a solid and comforting doctor-patient connection is essential. If nonpharmacological therapies are impractical for a patient, pharmacotherapy may be included in the therapeutic approach. Based on the most common symptom, a pharmacological treatment plan should be chosen, and a predetermined time frame should be set aside for dosage adjustments and efficacy assessments. Antibiotics like rifampin, peripheral opioid agonists, combined opioid agonists/antagonists, bile acid sequestrants, and antagonists of serotonin 5-hydroxytryptamine type 3 receptors are the primary treatment alternatives for people with irritable bowel syndrome with diarrhea (IBS-D). For individuals who are challenging to treat, lubiprostone and linaclotide should be saved as a last resort. Bulking agents and osmotic laxatives are the first line of therapy for irritable bowel syndrome leading to constipation (IBS-C). Because it may be affected by probiotics, prebiotics, synbiotics, and fecal microbiota transplantation, the gastrointestinal microbiota's role is an exciting research area. An updated summary of the most current developments in IBS treatment is provided in this review [13].

The clinical appearance of IBS varies significantly between people and even within the same individual over time. It is a widespread condition. Numerous symptoms, including bloating, constipation, diarrhea, and discomfort, might be signs of other gastrointestinal disorders, some of which have higher fatality rates, like people with conditions such as colorectal cancer or celiac disease. It leads to a severe problem for the clinician: how to look into a patient who seems to have IBS but whom you have a sneaking suspicion could be something else? Could ' something serious' go unnoticed? This brief overview offers an evidence-based solution to these perplexing queries and a helpful manual for identifying alternate diagnoses in patients exhibiting symptoms similar to IBS. A patient's differential diagnosis for IBS-like symptoms can be significantly reduced using clinical findings, patient demographics, and the clinical setting. They might enable an accurate IBS diagnosis. Separation from celiac and inflammatory bowel disease has been made much easier by developing noninvasive serological and stool testing. Microscopic colitis is something to think about for elderly female diarrhea patients. Although the significance of bile acid diarrhea is highlighted, the relationship between small intestine bacterial overgrowth and diarrhea-predominant IBS is still unclear. A focused and targeted examination will be made easier with careful attention to detail in the clinical evaluation of the person exhibiting IBS-like symptoms. Widely accessible fecal and serological testing, where appropriate, will support the diagnosis by ruling out alternative possibilities. The clinical presentation and circumstances should determine if more intrusive testing is necessary, with those with severe diarrhea often having a lower threshold for intervention [14].

Individuals suffering from IBS could have psychological effects since patients with inflammatory bowel disease frequently report having symptoms similar to IBS. Still-present inflammation has not been considered a possible cause in prior assessments of the scope of this problem. The prevalence of IBS-like symptoms in patients with inflammatory bowel disease (IBD) during remission is a significant concern. Studies suggest that a considerable proportion of individuals with IBD experience symptoms resembling those of IBS, such as abdominal pain and altered bowel habits, even when their inflammatory disease activity is low. Prevalence rates vary from approximately 20% to 50% or higher. Understanding and addressing these symptoms are crucial for optimizing patient care and improving the quality of life in this population [15].

The symptoms of IBS, a chronic, recurrent illness, include changed bowel patterns and pain or discomfort in the abdomen. Its complex pathophysiology necessitates efficient therapy since visceral hypersensitivity and impaired intestinal motility are the main contributors. Voltage-gated calcium channels are crucial for neuronal transmission and are involved in smooth muscle contraction and endocrine secretion. For many years, antispasmodics have been used to treat IBS. Alverine citrate is a selective 5-HT1A receptor antagonist and a spasmolytic that reduces the susceptibility of smooth muscle contractile proteins to calcium. In a sizable placebo-controlled experiment, alverine and simethicone successfully reduced pain and discomfort in the abdomen. A musculotropic substance called mebeverine effectively inhibits intestinal peristalsis. Mebeverine has demonstrated beneficial benefits for IBS symptom management in non-placebo-controlled trials; however, mebeverine did not outperform a placebo in recent placebo-controlled research. As an antimuscarinic, tachykinin NK2 receptor antagonist, and L-type calcium channel blocker, otilonium bromide is poorly absorbed from the GI tract. In IBS patients' placebo-controlled trials, polonium significantly decreased discomfort and enhanced changes in feces. Pinaverium bromide is an L-type calcium channel blocker located in the GI tract. Patients with IBS experience fewer bowel issues due to improved motility abnormalities in the pinaverium. In a placebo-controlled study, non-specific antispasmodics such as phenoglucinol and trimethylphloroglucinol helped IBS patients feel less discomfort. Excellent safety profiles are seen in antispasmodics. T-type calcium channel blockers are promising candidates for developing innovative therapeutic drugs to treat IBS since they can eliminate visceral hypersensitivity in animal models [16]. Both colonic diverticula and IBS are frequent symptomatic disorders in the West; nevertheless, the links between diverticula and IBS-type symptoms have been the subject of significant discussion. When diverticulitis, diverticular hemorrhage, and diverticulitis-related consequences, including stricture and fistula, are excluded from clinical situations about diverticula, it becomes clear that several questions are at the center of the interaction between IBS and diverticula. Let's ask if symptomatic uncomplicated diverticular disease (SUDD) and IBS are the same. Stated differently, is SUDD in a person with diverticula just like IBS? Consecutive diverticula and IBS are inevitable, but there are indications that SUDD may be slightly different, with more frequent and intense pain being its defining feature. Can an incident of acute diverticulitis cause the de novo development of IBS? This is similar to the interactions between IBS and inflammatory bowel or celiac disease. Nowadays, there is evidence for its incidence from pathophysiology and epidemiology [17].

Constant stomach discomfort and diarrhea are the hallmarks of post-infectious IBS (PI-IBS), which usually occurs after an episode of infectious gastroenteritis. Irritable bowel syndrome with leading diarrhea (IBS-D) underlying causes are unknown, although PI-IBS offers a mechanistic explanation. The analysis offers a current assessment of the pathogenesis, clinical characteristics, and therapeutic strategies of PI-IBS. The main characteristics of PI-IBS are dysregulated immune responses and cytokine release, which cause inflammation and malfunction in the gut. The diverse range of clinical severity can be attributed to several risk factors, including host genetic vulnerability, infectious agents, and disrupted brain-gut-microbiota interactions. While the prognosis is typically favorable, inflammation and symptoms may last a long time. The major strategies are symptomatic treatment with antidiarrheals, antispasmodics, 5HT3 antagonists, mesalamine, probiotics, and low-dose antidepressants. Nevertheless, a combination of medications that address the pathophysiology may be beneficial in certain challenging instances. There are several similarities between PI-IBS and IBS-D, including pathogenesis and therapeutic strategies [18]. Moreover, Table 1 shows the different categories of IBS.

**Table 1:** Categories in which irritable bowel syndrome is classified. Self created.

S. No.	Three primary subtypes of Irritable bowel syndrome
1.	Irritable bowel syndrome with leading constipation
2.	Irritable bowel syndrome with leading diarrhoea
3.	Irritable bowel syndrome with interchanging constipation and diarrhoea

Aetiology and pathophysiology

The chronic illness known as IBS is quite common and drastically lowers the value of life for those who suffer from it. The first-ever clinical guideline for treating IBS utilizing the Grading of Recommendations, Assessment, Development, and Evaluation (GRADE) system was developed by the American College of Gastroenterology in response to developments in diagnostic testing and treatment choices for individuals with IBS. After an extensive literature search, twenty-five clinically relevant issues were evaluated; nine questions concerned diagnostic tests, and sixteen concerned treatment choices. A modified Delphi technique was used to reach consensus, and we support the following recommendations based on GRADE methodology: To reduce the time it takes to start the right therapy, we recommend using a positive diagnostic approach rather than an excluding diagnostic approach. Serologic testing is advised to diagnose celiac disease in those with diarrhea and IBS. If someone has diarrheal symptoms and suspects they may have an inflammatory bowel disease, we advise testing for colonic calprotectin to rule out the condition. To help individuals with IBS have better overall symptoms, we advise a restricted trial of a low-fermentable oligosaccharides, disaccharides, monosaccharides, and polyols (FODMAP) diet. To treat constipation-related global IBS symptoms, we advise using guanylate cyclase activators and chloride channel activators. Rifaximin is suggested as a treatment for the overall IBS symptoms associated with diarrhea. For the treatment of overall IBS symptoms, we recommend gut-directed psychotherapy. The guidelines provide further claims and details about diagnoses, medications, dosages, and treatment duration [19].

The mechanism of IBS, a functional gastrointestinal ailment distressing many people, is still unclear but manifests as persistent stomach pain. Additionally, there currently need to be more studies on the effective combination of prebiotics and probiotics, or synbiotics, as a treatment for IBS. The critical role of probiotics in IBS has been identified thanks to technological advancements. On the other hand, the present paper centers on a comprehensive evaluation of the diverse pathophysiologic mechanisms through which probiotics, prebiotics, and synbiotics improve the symptoms of IBS. These mechanisms involve a variety of multifaceted domains, including neurological disease (microbiota-gut-brain axis interaction and co-morbidities) and digestive disorders (microbiota modification, modification of gut barrier function, heightened abdominal sensitivity, and gastrointestinal dysmotility). Furthermore, the research on the processes behind the therapeutic benefits of prebiotics and synbiotics for people with IBS is summarised in this analysis. It covers clinical studies on the efficacy and outcomes of synbiotic therapies for IBS patients [20]. A persistent functional gastrointestinal illness called IBS is typified by stomach pain brought on by changing bowel habits or passing gas during the elimination process. The gut microbiota has a mutualistic connection with the host, performing the duties of an actual organ, protecting the host from infections, and gaining additional energy and nutrients from the diet. Specific alterations in the makeup of this microbiota appear to be crucial to the pathophysiology of IBS. Diet has a well-established ability to modify the makeup of the gut microbiota. It is yet unknown, however, how specific dietary strategies are beneficial for IBS patients. The low-FODMAPS diet may alter the gut microbiota and hence affect the occurrence of gastrointestinal symptoms. This analysis examined how several dietary regimens (such as conventional nutritional advice, a low-FODMAP diet, a gluten-free diet [GFD], etc.) affected the intestinal microbiota changes and symptoms of both IBS-D patients and fit individuals. For people with IBS-D, there is not yet a perfect diet plan. Nonetheless, it becomes imperative to consider the impact of various dietary approaches on the makeup of the intestinal microbiota to establish an effective management plan for this functional illness [21].

Maintaining homeostasis is significantly aided by the gut-brain axis. Numerous internal and external variables affect signaling along this axis, adjusting how the central nervous and gastrointestinal systems operate. Recently, the notion of a microbiota-gut-brain axis has been developed, and the microbiome's significance as a significant player in influencing gut-brain signaling has emerged. This analysis focuses on the function of this axis in controlling the operation of the enteric and central nervous systems and how this may affect illnesses, including IBS, mood disorders, and affective disorders. We study the shared molecular frameworks underlying these illnesses, particularly the involvement of the neurotransmitter serotonin in the brain and gastrointestinal system. In summary, while research on animals has shown promising results, further development is required to transform these discoveries into beneficial diagnostic and therapeutic outcomes for human populations [22].

Risk factors and triggers

Significant challenges for the clinician arise from interpreting and managing symptoms that are so distressing to the patient, such as irritable bowel-like symptoms in patients with inflammatory bowel disease who appear to be in remission. The pathogenesis of these symptoms is still a subject of scientific controversy. Although anxiety is recognized as a cause of IBS, these symptoms generally meet the Rome IV criteria. However, new research has revealed that many individuals display mild inflammatory alterations. Such data raise whether these symptoms represent an active but subclinical type of IBD or "true" IBS superimposed on IBD. Although previous research was inevitably unable to identify subclinical inflammation, it is also clear that the occurrence of IBS-like symptoms in IBD patients is still higher than anticipated despite the use of sensitive biomarkers for inflammation, such as calprotectin and lactoferrin, which are supported by pan-endoscopy and biopsy to rule out ongoing inflammatory activity in its most subtle form [23].

Many times, gastrointestinal distress is thought to be triggered by eating for patients with IBS. Several variables, such as mast cell activation, nocebo and placebo response, altered gut microbiota, and malabsorption and fermentation of dietary substrates, are responsible for GI symptoms. Given the variability of symptoms and the likelihood of maladaptive eating behaviors in IBS patients, nutritional therapies must be tailored to each person. The low fermentable, oligo-, di-mono-saccharide, and polyol diet offers the most substantial evidence for effectiveness in managing symptoms, despite the wide range of therapies offered to people with IBS [24]. Irregular defecation patterns and repeated episodes of stomach discomfort characterize a chronic functional gastrointestinal illness called IBS. It impacts 5-10% of the wider population, contingent upon the criteria used, and significantly impacts quality of life. Most IBS patients report that eating particular foods like high-FODMAP foods (like onions and wheat), spicy foods, high-fat foods, caffeine, alcohol, carbonated beverages, artificial sweeteners, and dairy products for those with lactose intolerance causes their symptoms, particularly stomach pain, to start or worsen. This raises the question of what part food plays in the pathophysiology of IBS. Here, we outline the many risk factors for IBS and provide a general summary of how food might cause the condition, distinguishing between immunological and non-immune responses to food. In conclusion, we provide new research that reveals an immune-mediated mechanism behind food-induced discomfort in individuals with IBS. In individuals with IBS, certain foods can trigger discomfort through an immune-mediated mechanism. This involves the gut immune system reacting to specific food antigens, leading to inflammation, heightened gut sensitivity, and altered motility. Dysbiosis, or imbalance in the gut microbiota, may exacerbate this response. Understanding this mechanism could guide targeted dietary interventions or immunomodulatory therapies to alleviate symptoms and improve the quality of life for those with IBS [25].

Impact on psychological health

Psychological stress is a crucial factor in developing IBS. Clinical and experimental data show IBS, including an irritable brain [26]. In this analysis, we offer thorough methods of therapeutic therapy and address the possible involvement of psychological stress in the pathophysiology of IBS. Psychological stress profoundly impacts intestinal function, influencing sensitivity, motility, secretion, and permeability. These effects are mediated through the bidirectional communication pathways known as the gut-brain and brain-gut axes. Stress triggers changes in the central nervous system, peripheral neurons, gut microbiota, and mucosal immune activation, leading to alterations in gastrointestinal physiology. This complex interplay between the brain and gut highlights the importance of understanding the mind-body connection in gastrointestinal health. It underscores the potential for targeted interventions to alleviate stress-related gastrointestinal symptoms [26]. The underlying mechanism is closely linked to changes in the central nervous system, peripheral neurons, gastrointestinal microbiota, and mucosal immune activation. Stress-induced changes in neuro-endocrine-immune pathways influence the gut-brain and microbiota-gut-brain axes, leading to exaggeration or flare-ups of IBS symptoms. Stress is a trigger for IBS symptoms. Consequently, controlling stress and stress-related reactions should be the primary goal of treating IBS. These days, pharmacological and non-pharmacological methods that address stress-related changes - such as antidepressants, antipsychotics, various medications, inhibitors of 5-HT synthesis, selective reuptake inhibitors, and particular agonists or antagonists of 5-HT receptors - have demonstrated a vital role in the treatment of IBS. It is vital to handle IBS holistically [27].

According to national recommendations, psychological therapy should be considered while addressing IBS. Still, there have not been many head-to-head trials, so their relative effectiveness is unknown. Several psychological therapies are effective for IBS, yet none is better. The most substantial body of research supports the effectiveness of CBT-based therapies and gut-directed hypnosis over the long run [28]. There is a significant prevalence of psychological comorbidity in IBS. According to recent studies, anxiety, sadness, and visceral hypersensitivity have all been linked to gut bacteria. Additionally, an increased responsiveness of the illness to mental trauma has been reported. A few clinical investigations have tried to pinpoint dysbiosis characteristics in IBS patients. Human research on the correlation between gut microbiota and anxiety is rare, despite the significant correlations in animal studies. They are reviewing the most critical research on the gut microbial correlates of psychological and clinical characteristics of IBS, such as sadness, anxiety, and stress [29]. The underlying cause of IBS, a persistent functional bowel illness, is believed to be a malfunction in brain-gut communication. Centrally acting medications, such as antidepressants and psychosocial treatments, could work well. Patients with IBS can effectively reduce their symptoms by using antidepressants. Although there are gaps in the quality of the data and a potential overestimation of treatment benefits, psychological therapies also seem to be successful treatments for IBS [30].

Diet

If conventional lifestyle and nutritional advice for IBS are ineffective, a diet low in fermentable oligosaccharides, disaccharides, monosaccharides, and polyols is advised. Although some randomized controlled trials have examined how a low-FODMAPS diet affects specific IBS symptoms, a recent systematic evaluation has yet to be conducted. Since individual trials have looked at various alternative or control interventions, the best comparator needs to be clarified. To address these issues, we conducted a network-critical review. A low-FODMAPS diet came out on top across all endpoints examined in a network analysis. Nevertheless, most studies focused on secondary or tertiary treatment and did not examine how reintroducing and customizing FODMAPs affected symptoms [31]. IBS presents several difficulties for patients and doctors. IBS is difficult to manage and get good outcomes for various reasons, including its uncertain pathophysiology with several paths to investigate, bothersome symptoms that impair quality of life, and numerous subtypes of the disorder. Options for therapy begin with lifestyle guidance, go on to non-pharmaceutical approaches, and then address traditional approaches. Pharmacological therapy alternatives are not taken into consideration in this analysis. Consensus groups and meta-analyses have shown that while there are significant cultural and regional differences and variances in the intensity of recommendations, general guidelines are the same. Based on pertinent information, dietary modifications, probiotics, and fibers can be considered non-pharmaceutical therapies that coexist in different regimens to treat IBS symptoms [32]. Foods rich in highly fermentable oligo-, di-, and monosaccharides, polyols, and gluten have been linked to exacerbating IBS symptoms. It is still unclear, though, how dietary restrictions affect IBS symptoms. This study aimed to do a comprehensive analysis and critical review of randomised controlled trials (RCTs) that examined the effectiveness of exclusion diets (low FODMAP) and GFD in individuals with IBS [33]. Table 2 shows the summary of all articles included in this review relating to IBS.

**Table 2:** Summary of all articles included in this review. Self created.

Authors	Year	Findings
Haller and Scarlata [1]	2021	Individualization of nutritional therapy is necessary due to the variability of symptoms and the likelihood of maladaptive eating behaviors in IBS patients.
Dionne et al. [2]	2018	The data supporting the effectiveness of a low-FODMAPS diet in easing IBS symptoms could be more robust.
Ford et al. [3]	2019	Patients with IBS can effectively reduce their symptoms by using antidepressants.
Black et al. [4]	2020	The most comprehensive body of research supported the effectiveness of CBT-based therapies and gut-directed hypnosis over the long run.
Alamo and Quigley [5]	2019	Despite a reexamination of the link, the status of simple diverticular illness and IBS is still unknown.
Lee et al. [6]	2017	PI-IBS and IBS-D share a pathogenesis and management strategy and numerous common traits.
Hussein et al. [7]	2022	Food-mediated VHS is attributed to a local immunological reaction to food, which remarkably expands the pool of potential therapeutic therapies for IBS and associated illnesses.
Radovanovic-Dinic et al. [8]	2018	While some patients benefit significantly from non-pharmacological therapy, others also need it.
Fairbrass et al. [9]	2020	Depending on how remission was characterized, the prevalence of symptoms consistent with IBS differed between IBD patients.
Margolis et al. [10]	2021	The comprehension of the MGB axis in preclinical models of human brain diseases and the possible application of these discoveries to patients have advanced significantly.
Quigley [11]	2021	A focused and selected study will be made possible by careful attention to detail in the clinical examination of the person with IBS-like symptoms.
Koloski et al. [12]	2020	The brain-gut-immune-microbe axis is examined in light of promising findings to explain the causal relationship between psychological variables and FGIDs in some people.
Lacy et al. [13]	2021	Trained GRADE methodologists examined the literature pertinent to these 25 fundamental topics to evaluate the evidence quality and offer each recommendation's strength in this first-ever IBS Clinical Guideline.
Simrén et al. [14]	2013	One challenge for the future is to better define the function of intestinal microbiota in the development and pathophysiology of FBD.
Brenner et al. [15]	2023	Careful patient evaluation, including risk assessment and mental health screening, should inform the recommendation of digital BGBTs.
Altomare et al. [16]	2021	To determine, in these individuals, specific nutritional methods that can help alleviate symptoms by modifying the intestinal microbiota, further research has to be done on the association between dietary practices and the makeup of the gut microbiota.
Lackner [17]	2020	CBT has demonstrated efficacy when administered in-person, over the phone, online, or with limited therapist interaction; therapy improvements last after therapy is completed, in contrast to drugs.
Simon et al. [18]	2021	The effects of synbiotic delivery on their probiotic component, greater focus should be placed on anticipating the patients' probiotic reaction.
Rey et al. [19]	2017	Linaclotide has been shown in clinical trials to alleviate a broad range of IBS symptoms in individuals with IBS-C, such as constipation, bloating, and abdominal discomfort.
Islam et al. [20]	2022	Irritable bowel syndrome and fibromyalgia sufferers report better results from mind-body therapies, particularly CBT and yoga.
Algera et al. [21]	2023	It is anticipated that the delivery of psychological treatments—which now appear to be just as successful as many pharmaceutical treatment options—will advance with the application of contemporary communication technologies.
Ford et al. [22]	2020	Over the past ten years, a growing body of innovative medications has been developed due to an increased understanding of the pathophysiology of irritable bowel syndrome.
Marynowski et al. [23]	2015	There is very little evidence that environmental pollution has a role in the onset and course of IBS.
Moser et al. [24]	2018	The most significant research on the gut microbiota correlates with irritable bowel syndrome's psychological and clinical characteristics, such as sadness, anxiety, and stress.
Qin et al. [25]	2014	Managing stress and stress-induced reactions should be a significant focus of IBS treatment.
Padhy et al. [26]	2015	The disorder substantially impairs the quality of life, and the healthcare costs are high.
Ghoshal [27]	2020	Current data does not support the generalization that psychological illnesses are exclusively psychogenic, even if psychological problems are significant comorbid conditions in a subgroup of patients with FGID, including IBS.
Galica et al. [28]	2022	Although a low FODMAP diet may not be simple, the benefits accrue with time.
Black et al. [29]	2022	Studies that examined the impact of reintroducing and customizing FODMAPs on symptoms were primarily conducted in secondary or tertiary care settings.
Camilleri and Ford [30]	2017	Treating the patient's prominent, or most bothersome, symptom is still the primary goal of IBS treatment.
Annaházi et al. [31]	2014	Antispasmodics without cardiovascular effects—such as phloroglucinol, alverine citrate, mebeverine, otilonium bromide, and pinaverium bromide—are frequently utilized in treating IBS.
Edgington-Mitchell [32]	2016	Protease balance between proteases and their inhibitors is essential for preserving normal gut homeostasis since evidence for how proteases contribute to the pathophysiology of gastrointestinal disorders such as IBD, IBS, and CRC is mounting.
Bonetto et al. [33]	2021	The importance of the fecal microbiota in IBS has drawn more attention, and probiotics, prebiotics, and synbiotics are standard treatment options.

## Conclusions

In primary care, patients with irritable bowel syndrome are often seen. Patients with IBS in primary care tend to be young females and have milder symptoms than those in secondary care. This is only the case for some symptoms; non-abdominal issues, for instance, are similarly reported in the two groups. Rome II criteria, which are symptom-based criteria universally agreed upon, can be used to diagnose the disease safely. In very few cases, further diagnostic testing is required to confirm the diagnosis. With enough confidence and education, many general practitioner IBS patients may be treated, often without the need for extra pharmaceutical therapy. The overwhelming majority of patients for whom initial treatments for IBS, along with treatment of concurrent psychological disorders, are insufficient, could benefit from improvements in the diagnosis and management of symptoms due to advancements in the detection of specific disorders as causes of individual symptoms in the "IBS spectrum." The study highlights a significant association between IBS and psychiatric disorders, particularly depression, among participants. However, it reveals a concerning gap in the identification and evaluation of these comorbid conditions by primary physicians, with only a minority of individuals with IBS undergoing thorough mental health assessments. The authors stress the importance of addressing psychiatric disorders alongside IBS to optimize patient care and prevent unnecessary medical interventions. They recommend routine screening for psychiatric disorders at the time of IBS diagnosis to ensure early detection and appropriate management, ultimately improving both gastrointestinal symptoms and overall mental well-being in affected individuals.
